# Type of vegetarian diet, obesity and diabetes in adult Indian population

**DOI:** 10.1186/1475-2891-13-89

**Published:** 2014-09-05

**Authors:** Sutapa Agrawal, Christopher J Millett, Preet K Dhillon, SV Subramanian, Shah Ebrahim

**Affiliations:** South Asia Network for Chronic Disease, Public Health Foundation of India, Fourth Floor, Plot no 47, Sector 44, Gurgaon (Haryana) 122002 New Delhi, India; Department of Primary Care and Public Health, School of Public Health, Imperial College, London, UK; Department of Society, Human Development and Health, Harvard School of Public Health, Harvard University, Boston, USA; Department of Non-communicable Disease Epidemiology, London School of Hygiene and Tropical Medicine, London, UK

**Keywords:** Vegetarian diets, Diabetes, Obesity, Men, Women, NFHS-3, India

## Abstract

**Background:**

To investigate the prevalence of obesity and diabetes among adult men and women in India consuming different types of vegetarian diets compared with those consuming non-vegetarian diets.

**Methods:**

We used cross-sectional data of 156,317 adults aged 20–49 years who participated in India’s third National Family Health Survey (2005–06). Association between types of vegetarian diet (vegan, lacto-vegetarian, lacto-ovo vegetarian, pesco-vegetarian, semi-vegetarian and non-vegetarian) and self-reported diabetes status and measured body mass index (BMI) were estimated using multivariable logistic regression adjusting for age, gender, education, household wealth, rural/urban residence, religion, caste, smoking, alcohol use, and television watching.

**Results:**

Mean BMI was lowest in pesco-vegetarians (20.3 kg/m^2^) and vegans (20.5 kg/m^2^) and highest in lacto-ovo vegetarian (21.0 kg/m^2^) and lacto-vegetarian (21.2 kg/m^2^) diets. Prevalence of diabetes varied from 0.9% (95% CI: 0.8-1.1) in person consuming lacto-vegetarian, lacto-ovo vegetarian (95% CI:0.6-1.3) and semi-vegetarian (95% CI:0.7-1.1) diets and was highest in those persons consuming a pesco-vegetarian diet (1.4%; 95% CI:1.0-2.0). Consumption of a lacto- (OR:0.67;95% CI:0.58-0.76;p < 0.01), lacto-ovo (OR:0.70; 95% CI:0.51-0.96;p = 0.03) and semi-vegetarian (OR:0.77; 95% CI:0.60-0.98; p = 0.03) diet was associated with a lower likelihood of diabetes than a non-vegetarian diet in the adjusted analyses.

**Conclusions:**

In this large, nationally representative sample of Indian adults, lacto-, lacto-ovo and semi-vegetarian diets were associated with a lower likelihood of diabetes. These findings may assist in the development of interventions to address the growing burden of overweight/obesity and diabetes in Indian population. However, prospective studies with better measures of dietary intake and clinical measures of diabetes are needed to clarify this relationship.

## Background

Studies from Western countries suggest that vegetarian diets may have a protective role against the development of obesity and diabetes [1–5]. The European Prospective Investigation Study (EPIC-Oxford) found that mean BMI was highest in meat-eaters, lowest in vegans, and intermediate in fish-eaters and vegetarians [6]. In the Nurses’ Health Study, intake of red meat and processed meats were associated with increased risk of diabetes [7]. In Seventh-day Adventist cohort studies initiated in the 1960s–1970s, diabetes was less prevalent in vegetarian than in semi-vegetarian (those who ate fish and poultry, but <1 time/wk)_ or non-vegetarian church-goers and processed meat eaters [5, 7–9]. These observational findings are also supported by experimental data which have shown that the selection of foods found in vegetarian diets may carry metabolic advantages for the prevention of type 2 diabetes [10].

India is experiencing an alarming increase in the prevalence of type 2 diabetes [10–15]. The resulting morbidity, economic costs, reduced quality of life, and risk for complications make preventive strategies imperative. The contribution of the Indian diet to the increasing prevalence of diabetes in the country is not well understood. Within this, there is little information on whether the vegetarian diet confers a similar protective effect against obesity and diabetes that have been demonstrated in western studies. This is an important question given the ongoing preponderance of vegetarianism in certain social and religious groups in India coupled with an increase in meat eating associated with growth in western-style diets in some section of the Indian society. Moreover, vegetarianism in India is associated with unique characteristics. It is usually a lifelong pattern and adherence crosses multiple generations; it generally comprises high consumption of whole grains, legumes, nuts and seeds and dairy with spices and seasonings unique to the Indian diet. Hence, the combination/or the pattern of vegetarian diet may yield different findings than similar studies conducted in the West and it is thus possible to assess dietary associations with chronic diseases which have been difficult in the West due to low frequency. This study uses data from the third National Family Health Survey (NFHS-3, 2005–06), a survey of 109,041 Indian households which collected information on a wide range of dietary, societal, lifestyle, and environmental determinants of morbidity and chronic ailments including diabetes [16]. The NFHS-3 provides a unique opportunity to examine associations between types of vegetarian diet and diabetes and obesity in a large, nationally representative sample. We hypothesized that exclusively vegetarian diets, such as vegan, lacto- or lacto-ovo vegetarian, are associated with a lower prevalence of diabetes and obesity compared with a non-vegetarian diet.

## Methods

### Data and study setting

We used cross-sectional data from India’s third National Family Health Survey (NFHS-3, 2005-06) conducted in 29 states which comprises more than 99% of the country’s population, but excluded the Union Territories. Details of survey objectives and survey methods including sampling frame and questionnaires are provided elsewhere [16]. Briefly, this survey was designed on the lines of the Demographic and Health Surveys (DHS) (available at http://www.measuredhs.com) that have been conducted in many developing countries since the 1980s and in India the survey was designed to provide estimates of key indicators (except HIV prevalence) for each state by urban and rural areas. The NFHS has been conducted in India for successive three rounds, each at an interval of 5 years. NFHS-3 is the most recent major national health survey in India that collected demographic, socioeconomic and health information from a nationally representative probability sample of 124,385 women (62.6%) aged 15-49 years and 74,369 men (37.4%) aged 15-54 years residing in 109,041 households. The data was obtained at the individual level by face-to-face interviews conducted in the respondents’ homes.

The NFHS-3 samples were geo-coded to the primary sampling unit, district, and state to which they belonged. A uniform multistage sampling strategy was adopted in all the states, with separate sampling in urban and rural areas. In rural areas, a two-stage sample was carried out using a list of villages from the 2001 census as the sampling frame. In the first stage, a stratified sample of villages was drawn with probability proportional to the size of the village. In the second stage, a random selection of households was drawn in each village from a complete list of households compiled during field visits carried out in each sampled village. In urban areas, a similar procedure was implemented beginning with a stratified random sample of municipal wards based on the 2001 census. Further, one census enumeration block (about 150–200 households) was selected from within the wards using probability proportional to size sampling frame. Finally, as in rural areas, field enumerators undertook a house listing operation in selected blocks and a random sample of households was made. In both rural and urban areas, 30 households were targeted for selection in each of the sampled units.

The overall household response rate in NFHS-3 was 98%. All women aged 15–49 years in selected households were invited to participate in the National Family Health Survey. Interviews were conducted in one of the 18 Indian languages in the respondent’s home and the response rates were 95% for women and 87% for men. During interviews, the weights and heights of survey respondents were measured and blood sample were drawn by trained field technicians using standardised measuring equipment designed for survey settings in developing country.

The analysis we present here is restricted to 156,317 sample population comprising of 99,574 women and 56,742 men aged 20-49 years living in the sample households. We excluded age below 20 years to avoid any cases of childhood diabetes for which the etiology and risk factors might be different. Age above 50 years is also excluded (for men only) for comparison purpose since information for women age above 50 years is not collected in the survey.

### Outcome evaluation

The survey asked participants the question, *‘Do you currently have diabetes?’*. However, neither data on physician-reported diagnosis of diabetes nor data on fasting blood glucose was available in the NFHS-3 to verify a self-report.

### Assessment of dietary intake

In NFHS-3, consumption of selected food item was assessed by asking, ‘*How often do you yourself consume the following food items: daily, weekly, occasionally or never?’* related to the consumption of milk or curd, pulses or beans, dark green leafy vegetables, fruits, eggs, fish, chicken or meat. Based on the frequency of consumption, vegetarian status [8, 17, 18] was categorized by defining vegans as subjects who reported never consuming animal products (chicken or meat, fish, eggs, milk or curd); lacto-vegetarian as those who reported consuming fruits, vegetables, pulses or beans, milk or curd, either daily, weekly or occasionally but no fish, eggs or chicken or meat; lacto-ovo vegetarian as those who reported consuming fruits, vegetables, pulses or beans, milk or curd, and or eggs either daily, weekly or occasionally but no fish or chicken or meat; pesco-vegetarian: who reported consuming fruits, vegetables, pulses or beans, milk or curd, and or eggs or fish either daily, weekly or occasionally but no chicken or meat; semi-vegetarian: who reported consuming fruits, vegetables, pulses or beans, animal products (chicken or meat, eggs, milk or curd) either daily, weekly or occasionally but no fish; non-vegetarian: who reported consuming fruits, vegetables, pulses or beans, animal products (chicken or meat, fish, eggs, milk or curd) either daily, weekly or occasionally.

### Other predictor variables and covariates

The survey collected information on demographic, socioeconomic factors, lifestyle factors and anthropometric measurements. Respondents were weighed using a solar powered digital scale (SECA 874 digital scale ^a^) with an accuracy of ±100 g [19]. Their height was measured using an adjustable wooden measuring board, specifically designed to provide accurate measurements (to the nearest 0.1 cm) [19]. Indian adult population standard [20–22] categories of Body Mass Index (BMI, kg/m^2^) were used: ≤18.5 kg/m^2^ (underweight); 18.5 to 22.9 kg/m^2^ (normal), 23.0 to 24.9 kg/m^2^ (overweight), and ≥25.0 kg/m^2^ (obese). The information on exposure to tobacco smoke was—yes–active smoking (person currently smokes) and no smoking (the person has never smoked). Information on past smoking was not available in the dataset. Use of alcohol was quantified as ever drinker (drinks taken almost every day or about once weekly or less than once weekly) and never drinker. Frequency of watching television (almost every day, at least once weekly, less than once weekly, not at all) was used as a measure of sedentary behavior. Other covariates in our analysis include: age categories (20–29, 30–39, 40–49 years); gender, education (no education, primary complete, middle complete, higher and above); religion (Hindu, Muslim, Christian, Sikh, Others); caste/tribe (scheduled caste, scheduled tribe, other backward class, others, missing caste); wealth quintiles (based on 33 assets and housing characteristics graded lowest, second, middle, fourth, highest); and place of residence (urban, rural). For detailed definition of some variables and items used for construction of the wealth index, see Table 1.

**Table 1:** **Sample distribution and percentage prevalence of diabetes among men (n = 56,742) and women (n = 99,574) age 20–49 years according to intake of specific food items in the national family health survey, India 2005-06**

Frequency of intake	Men			Women			Total		
	Total N [%]	Diabetes cases N [%]	χ^2^p value	Subjects N [%]	Diabetes cases N [%]	χ^2^p value	Total N [%]	Diabetes cases N [%]	χ^2^p value
Milk or curd			<0.0001			<0.0001			<0.0001
Daily	26307 [46.4]	391 [1.5]		40366 [40.5]	492 [1.2]		66673 [42.7]	883 [1.3]	
Weekly	11554 [20.4]	117 [1.0]		15071 [15.1]	138 [0.9]		26626 [17.0]	255 [1.0]	
Occasionally	14757 [26.0]	138 [0.9]		32918 [33.1]	302 [0.9]		47675 [30.5]	440 [0.9]	
Never	4114 [7.3]	74 [1.8]		11202 [11.3]	117 [1.0]		15317 [9.8]	191 [1.2]	
Pulses and beans			<0.0001			<0.0001			<0.0001
Daily	29863 [52.6]	437 [1.5]		52440 [52.7]	538 [1.0]		82303 [52.7]	975 [1.2]	
Weekly	21705 [38.3]	219 [1.0]		36597 [36.8]	360 [1.0]		58302 [37.3]	579 [1.0]	
Occasionally	4660 [8.2]	51 [1.1]		9663 [9.7]	131 [1.4]		14323 [9.2]	182 [1.3]	
Never	505 [0.9]	13 [2.6]		852 [0.9]	20 [2.3]		1357 [0.9]	33 [2.4]	
Green leafy vegetables			0.149			0.090			0.368
Daily	33982 [59.9]	453 [1.3]		64095 [64.4]	674 [1.1]		98076 [62.7]	1127 [1.1]	
Weekly	19270 [34.0]	231 [1.2]		28606 [28.7]	286 [1.0]		47876 [30.6]	517 [1.1]	
Never/ Occasionally	3480 [6.1]	35 [1.0]		6840 [6.9]	89 [1.3]		10321 [6.6]	125 [1.2]	
Fruits			<0.0001			<0.0001			<0.0001
Daily	7320 [12.9]	125 [1.7]		12789 [12.9]	206 [1.6]		20109 [12.9]	331 [1.6]	
Weekly	19368 [34.1]	255 [1.3]		26731 [26.9]	276 [1.0]		46099 [29.5]	531 [1.2]	
Occasionally	28484 [50.2]	296 [1.0]		56336 [56.6]	503 [0.9]		84820 [54.3]	800 [0.9]	
Never	1546 [2.7]	44 [2.8]		3631 [3.6]	63 [1.7]		5177 [3.3]	107 [2.1]	
Eggs			<0.0001			<0.0001			<0.0001
Daily	2931 [5.2]	56 [1.9]		3475 [3.5]	60 [1.7]		6405 [4.1]	115 [1.8]	
Weekly	20682 [36.5]	317 [1.5]		28778 [28.9]	363 [1.3]		49460 [31.6]	680 [1.4]	
Occasionally	19786 [34.9]	201 [1.0]		32635 [32.8]	287 [0.9]		52421 [33.5]	488 [0.9]	
Never	13330 [23.5]	146 [1.1]		34647 [34.8]	340 [1.0]		47977 [30.7]	486 [1.0]	
Fish			<0.0001			<0.0001			<0.0001
Daily	3706 [6.5]	90 [2.4]		6505 [6.5]	149 [2.3]		10211 [6.5]	240 [2.4]	
Weekly	14414 [25.4]	238 [1.7]		22070 [22.2]	304 [1.4]		36484 [23.3]	542 [1.5]	
Occasionally	21818 [38.5]	225 [1.0]		34242 [34.4]	264 [0.8]		56060 [35.9]	489 [0.9]	
Never	16782 [29.6]	167 [1.0]		36724 [36.9]	331 [0.9]		53506 [34.2]	498 [0.9]	
Chicken or meat			<0.0001			<0.0001			<0.0001
Daily	706 [1.2]	6 [0.9]		839 [0.8]	14 [1.7]		1545 [1.0]	20[1.3]	
Weekly	15609 [27.5]	269 [1.7]		21938 [22.0]	292 [1.3]		37548 [24.0]	561[1.5]	
Occasionally	26135 [46.1]	291 [1.1]		42222 [42.0]	423 [1.0]		68357 [43.7]	714[1.0]	
Never	14272 [25.2]	155 [1.1]		34537 [34.7]	320 [0.9]		48809 [31.2]	475[1.0]	

### Statistical analysis

As certain states and certain groups of respondents were oversampled, sample weights were used to restore the representativeness of the sample [16]. Descriptive statistics were calculated with the use of standard methods (such as frequencies and percentages) for the total sample (n = 156,317). Differences in proportions for categorical variables were tested using Pearson’s *χ*^2^ tests. Trend tests were also carried out scoring the variables in different categories by using likelihood ratio tests. Multivariable logistic regression models were used to estimate the odds ratios of types of vegetarian diet intake on self-reported diabetes after controlling for potential confounders and also examining the independent effects of other risk factors. Both unadjusted and adjusted models were constructed with 95% confidence intervals to account for potential confounders and mediators. Model 1 presents unadjusted results; Model 2 presents results adjusted for BMI, lifestyle factors and socio-demographic factors which may be confounders to exhibit any independent effect of vegetarian diet on diabetes prevalence; Model 3 presents results adjusted for all the above factors except BMI. Results are presented in the form of odds ratios (ORs) with 95% confidence intervals (95% CI). The estimation of confidence intervals takes into account the design effects due to clustering at the level of the primary sampling unit. Before carrying out the models, we tested for the possibility of multicolinearity between the variables. In the correlation matrix, all pair wise Pearson correlation coefficients are <0.5, suggesting that multicolinearity is not a problem. All the analysis including the logistic regression models were conducted using the SPSS statistical software package, version 19 (IBM SPSS Statistics, Chicago, IL, USA).

As the effect of type of vegetarian diet consumption on the prevalence of diabetes are likely to vary by sex, due to the large gender differences in nutritional status^b^ in India, the susceptibility to disease, and access to treatment and care in a developing country in general, an analysis was also carried out for men and women separately.

### Ethics statement

The data were analyzed anonymously, using publicly available secondary data, therefore no ethics review is required for this work. The National Family Health Survey was approved by the ethical review boards of the implementing agencies and the Indian government. Participation in the survey was totally voluntary. The survey obtained written informed consent from each respondent (men and women) before asking questions, and separately before obtaining height and weight measurements.

## Results

### Types of vegetarian diet consumption in India and states

The sample distribution and percentage prevalence of diabetes among men (n = 56,742) and women (n = 99,574) aged 20–49 years according to intake of specific food items in the National Family Health Survey, India 2005–06 is presented in Table 1. Table 2 gives the percentage consumption of different types of vegetarian diet among adult population (n = 156,317) age 20–49 years in India and states. Overall a majority (two-third- 64%) of the sample population eat a non-vegetarian diet either daily, weekly or at least occasionally where as one-fourth is lacto-vegetarian (Table 1). Other dietary patterns are followed by a relatively smaller percentage of Indian population: semi-vegetarian-5.2%, lacto-ovo vegetarian-3.2%, pesco-vegetarian-2.2% and vegan-1.6%. More than 80% of the population consume a non-vegetarian diet in northeastern region, in southern region (except the state of Karnataka), most of the states in eastern region (except Bihar), and the western state of Goa. More than half the population in the northern states of Punjab, Haryana, Rajasthan, and in the western state of Gujarat follow a lacto-vegetarian diet. One in five people in Jammu and Kashmir follow a semi-vegetarian diet (without fish) whereas one in ten people in Goa (11.8%) and Manipur (9.0%), 7.8% in Tripura, 7.2% in Orissa, 5.2% in Kerala and 4.1% in West Bengal consume pesco-vegetarian diet (dominated by fish). In the state of Delhi, one out of ten people is a lacto-ovo vegetarian where as the western states of Gujarat (4.9%) and Maharashtra (4.0%) have the highest percentages of vegans.

**Table 2:** **Percentage consumption of different types of diet among adult population age 20–49 years in India and states, NFHS-3, 2005-06**

India/States	Types of diet						Total N
	Vegan N [%]	Lacto-vegetarian N [%]	Lacto-ovo vegetarian N [%]	Pesco- vegetarian N [%]	Semi-vegetarian N [%]	Non-vegetarian N [%]	
**India**	2560 [1.6]	37797 [24.2]	5002 [3.2]	3446 [2.2]	8140 [5.2]	99372 [63.6]	156317
**Northern region**							
Jammu & Kashmir	9 [0.6]	276 [18.4]	18 [1.2]	9 [0.6]	297 [19.8]	891 [59.4]	1500
Himachal Pradesh	17 [1.8]	429 [45.6]	76 [8.1]	13 [1.4]	137 [14.6]	269 [28.6]	941
Punjab	138 [3.4]	2149 [52.3]	275 [6.7]	13 [0.3]	420 [10.2]	1112 [27.1]	4107
Uttaranchal	20 [1.6]	324 [26.6]	84 [6.9]	15 [1.2]	108 [8.9]	669 [54.8]	1220
Haryana	107 [3.5]	2099 [68.9]	205 [6.7]	6 [0.2]	148 [4.9]	482 [15.8]	3047
Delhi	43 [2.1]	645 [30.9]	222 [10.6]	25 [1.2]	192 [9.2]	963 [46.1]	2090
Rajasthan	236 [2.9]	5060 [62.1]	393 [4.8]	62 [0.8]	869 [10.7]	1528 [18.8]	8148
**Central region**							
Uttar Pradesh	264 [1.2]	8458 [37.7]	1227 [5.5]	336 [1.5]	835 [3.7]	11343 [50.5]	22463
Chhattisgarh	69 [2.1]	484 [14.5]	101 [3.0]	60 [1.8]	57 [1.7]	2574 [77.0]	3345
Madhya Pradesh	294 [3.1]	3975 [42.2]	479 [5.1]	223 [2.4]	463 [4.9]	3980 [42.3]	9414
**Eastern region**							
Bihar	50 [0.5]	1812 [17.3]	66 [0.6]	382 [3.6]	120 [1.1]	8037 [76.8]	10467
West Bengal	43 [0.3]	183 [1.4]	16 [0.1]	554 [4.1]	94 [0.7]	12548 [93.4]	13438
Jharkhand	49 [1.3]	214 [5.5]	37 [1.0]	80 [2.1]	81 [2.1]	3395 [88.0]	3856
Orissa	50 [0.8]	225 [3.8]	19 [0.3]	432 [7.2]	66 [1.1]	5168 [86.7]	5960
**Northeastern region**							
Sikkim	0 [0.0]	9 [9.6]	1 [1.1]	1 [1.1]	6 [6.4]	77 [81.9]	94
Arunachal Pradesh	0 [0.0]	2 [1.3]	1 [0.6]	2 [1.3]	2 [1.3]	152 [95.6]	159
Nagaland	0 [0.0]	1 [0.5]	0 [0.0]	1 [0.5]	2 [1.0]	204 [98.1]	208
Manipur	1 [0.3]	1 [0.3]	0 [0.0]	31 [9.0]	3 [0.9]	307 [89.5]	343
Mizoram	0 [0.0]	0 [0.0]	1 [0.7]	1 [0.7]	6 [4.3]	131 [94.2]	139
Tripura	1 [0.2]	4 [0.7]	1 [0.2]	46 [7.8]	2 [0.3]	536 [90.8]	590
Meghalaya	0 [0.0]	3 [0.8]	1 [0.3]	9 [2.3]	5 [1.3]	371 [95.4]	389
Assam	5 [0.1]	72 [1.6]	13 [0.3]	132 [3.0]	12 [0.3]	4135 [94.6]	4369
**Western region**							
Gujarat	400 [4.9]	4546 [55.6]	342 [4.2]	159 [1.9]	399 [4.9]	2330 [28.5]	8176
Maharashtra	643 [4.0]	3614 [22.7]	529 [3.3]	1.35 [0.8]	912 [5.7]	10068 [63.3]	15901
Goa	3 [1.2]	10 [3.9]	2 [0.8]	30 [11.8]	2 [0.8]	207 [81.5]	254
**Southern region**							
Andhra Pradesh	45 [0.4]	579 [4.7]	222 [1.8]	78 [0.6]	1129 [9.1]	10299 [83.4]	12352
Karnataka	41 [0.4]	2126 [22.2]	385 [4.0]	134 [1.4]	979 [10.2]	5932 [61.8]	9597
Kerala	11 [0.2]	81 [1.8]	37 [0.8]	234 [5.2]	54 [1.2]	4045 [90.7]	4462
Tamil Nadu	21 [0.2]	416 [4.5]	249 [2.7]	243 [2.6]	740 [8.0]	7619 [82.0]	9288

### Distribution of self reported diabetes cases and diabetes prevalence by study covariates

Among those who reported diabetes, three out of five were aged between 40–49 years, a majority (59%) were women, two out of five had a secondary education, three-fourth follow Hindu religion, two out of five belonged to general caste, two out of five belonged to household with a highest wealth status, a majority don’t smoke or drink alcohol, more than half the participants watched TV almost every day and a third were obese (Table 3).

**Table 3:** **Percentage distribution of participants by self reported diabetes status and prevalence of diabetes according to non-dietary variables, India NFHS 2005-06**

Characteristics	Total participants N [%]*	Diabetes cases		***χ*** ^2^P values	Diabetes prevalence N [%]	***χ*** ^2^P values
		Reported N [%]	Not reported N [%]			
N [%]	156,317	1,769 [1.1]	154,501 [98.9]			
Age				<0.0001		<0.0001
20-29 y	66,038 [42.2]	204 [11.5]	65807 [42.6]		0.3	
30-39 y	52,567 [33.6]	520 [29.4]	5203833.7]		1.0	
40-49 y	37,711 [24.1]	1045 [59.1]	36656 [23.7]		2.8	
Sex				<0.0001		<0.0001
Men	56,742 [36.3]	994 [40.7]	73367 [36.3]		1.3	
Women	99,574 [63.7]	1096 [59.3]	123244 [63.7]		0.9	
Education^a^				<0.0001		<0.0001
No education	56,720 [36.3]	529 [27.3]	63709 [36.4]		0.8	
Primary	24,493 [15.7]	350 [17.1]	30593 [15.7]		1.1	
Secondary	58,448 [37.4]	909 [42.6]	84284 [37.3]		1.1	
Higher and above	16,639 [10.6]	302 [13.0]	18001 [10.6]		1.7	
Caste/tribe^b^				<0.0001		<0.0001
Scheduled caste	29831 [18.5]	350 [17.2]	36736 [18.5]		0.9	
Scheduled tribe	12734 [8.1]	75 [3.1]	16105 [8.2]		0.5	
Other backward class	60977 [39.0]	728 [35.3]	77187 [39.0]		0.9	
General	48854 [31.3]	840 [39.9]	60489 [31.2]		1.4	
Missing caste	4821 [3.1]	90 [4.6]	5450 [3.1]		1.6	
Religion^c^				<0.0001		<0.0001
Hindu	127375 [81.5]	1616 [77.3]	159511 [81.5]		1.0	
Muslim	19781 [12.7]	311 [15.1]	25806 [12.6]		1.2	
Christian	3816 [2.4]	86 [4.2]	4657 [2.4]		1.8	
Sikh	2845 [1.8]	48 [2.1]	3534 [1.8]		1.3	
Others	2500 [1.6]	28 [1.2]	3104 [1.6]		0.8	
Wealth quintiles^d^				<0.0001		<0.0001
Lowest	26389 [16.9]	171 [8.0]	33269 [17.0]		0.5	
Second	28751 [18.4]	270 [13.6]	36780 [18.4]		0.7	
Middle	31232 [20.0]	272 [13.1]	39975 [20.1]		0.7	
Fourth	33560 [21.5]	490 [24.2]	42082 [21.4]		1.2	
Highest	36385 [23.3]	887 [41.0]	44505 [23.1]		2.0	
Place of residence				<0.0001		<0.0001
Urban	54134 [34.6]	1068 [50.8]	66879 [34.4]		1.6	
Rural	102183 [65.4]	1022 [49.2]	129732 [65.6]		0.8	
Body Mass Index (kg/m^2^)^e^				<0.0001		<0.0001
≤18.5 kg/m^2^	46021 [30.9]	694 [14.6]	85097 [31.1]		0.8	
18.5-22.9 kg/m^2^	67836 [45.5]	295 [33.2]	64754 [45.7]		0.5	
23.0-24.9 kg/m^2^	15089 [10.1]	347 [16.4]	16537 [10.1]		2.1	
≥25.0 kg/m^2^	20050 [13.5]	691 [35.8]	20858 [13.2]		3.2	
Current Tobacco smoking				<0.0001		<0.0001
No	133160 [85.2]	1736 [83.7]	170086 [85.2]		1.0	
Yes	23156 [14.8]	355 [16.3]	26525 [14.8]		1.3	
Alcohol consumption				0.015		<0.0001
Never	133067 [85.1]	1705 [83.3]	170416 [85.1]		1.0	
Ever	23250 [14.9]	385 [16.7]	26177 [14.9]		1.4	
Frequency of watching TV				<0.0001		<0.0001
Not at all	45916 [29.4]	403 [20.7]	55562 [29.5]		0.7	
Less than once a week	21859 [14.0]	232 [10.8]	27442 [14.0]		0.8	
At least once a week	20033 [12.8]	257 [12.0]	26059 [12.8]		1.0	
Almost everyday	68480 [43.8]	1198 [56.5]	87516 [43.7]		1.4	

*Total participants varies slightly for individual variables depending on the number of missing values.

^a^Education: illiterate (0 years of education), literate but less than middle school complete (1–5 years of education), middle school complete (6–8 years of education), high school complete or more (9+ years of education).

^b^Scheduled castes and scheduled tribes are identified by the Government of India as socially and economically backward and needing protection from social injustice and exploitation. Other backward class is a diverse collection of intermediate castes that were considered low in the traditional caste hierarchy but are clearly above scheduled castes. Others is thus a default residual group that enjoys higher status in the caste hierarchy.

^c^Others include Buddhist, Jain, Jewish, Zoroastrian.

^d^The wealth index is based on following assets in the household: household electrification, type of windows, drinking water source, type of toilet facility, type of flooring, material of exterior walls, type of roofing, house ownership, ownership of a bank or post office account, and ownership of a mattress, a pressure cooker, a chair, a cot/bed, a table, an electric fan, a radio/transistor, a black and white television, a colour television, a sewing machine, a mobile telephone, any other telephone, a computer, a refrigerator, a watch or clock, a bicycle, a motorcycle or scooter, an animal-drawn cart, a car, a water pump, a thresher, and a tractor.

^e^Women who were pregnant at the time of the survey or women who had given birth during the two months preceding the survey were excluded from these measurements.

The overall prevalence of diabetes was significantly higher (p < 0.0001) among men (1.3%) than among women (0.9%) (Table 3). Significant associations between age and diabetes prevalence were observed, diabetes being more prevalent (2.8%) in the highest age group (40–49 years). Diabetes prevalence increased according to household wealth status and was almost double in urban population compared with their rural counterparts (1.6 vs 0.8) and highest among those with a higher secondary and above education (all p < 0.0001). Prevalence of diabetes was also higher among those who were currently smoking tobacco (1.3%) or ever consumed alcohol (1.4%), who were either overweight (2.1%) or obese (3.2%) and those who watched television almost every day (1.4%).

### Prevalence of diabetes and obesity according to types of vegetarian diet consumption

Table 4 gives the unadjusted prevalence of diabetes and obesity by types of vegetarian diet consumption. No apparent trend in diabetes prevalence based on type of vegetarian diet was found (p for trend = 0.002). Prevalence of diabetes varied from 0.9% (95% CI:0.8-1.1) each in lacto-vegetarian, lacto-ovo vegetarian (95% CI:0.6-1.3) and semi-vegetarian (95% CI:0.7-1.1) to 1.0% in vegan (95% CI: 0.6-1.7), 1.2% (95% CI:1.1-1.3) in non-vegetarian and highest in pesco-vegeterian diets (1.4%; 95% CI:1.0-2.0). The range between the lowest and highest BMIs for all groups were reasonably low (less than 1 kg/m^2^). Mean BMI was 20.3 kg/m^2^ in pesco-vegetarians and 20.5 kg/m^2^ in vegans, 20.6 kg/m^2^ in semi-vegetarians, 20.7 kg/m^2^ in non-vegetarians, 21.0 kg/m^2^ in lacto-ovo vegetarians and 21.2 kg/m^2^ in lacto-vegetarians. For BMIs ≥23 kg/m^2^, the prevalence of diabetes was 1.7% in lacto-ovo vegetarians, 2.0% in semi-vegetarians, 2.1% in lacto-vegetarians, 2.6% in pesco-vegetarians, 2.8% in vegans, and 2.9% in non-vegetarians (data not shown). For BMIs ≥30 kg/m^2^, the prevalence of diabetes was 2.1% in lacto-ovo vegetarians, 3.7% in lacto-vegetarians, 3.8% in semi-vegetarians, 5.2% in vegans, 5.3% in pesco-vegetarians and 5.4% in non-vegetarians (data not shown).

**Table 4:** **Unadjusted prevalence (% with CI) of diabetes and obesity according to types of vegetarian diet consumption in adult Indian population (n = 156,317) aged 20–49 years, NFHS 2005-06**

Characteristics	Type of diets						P for trend values*
	Vegan	Lacto-vegetarian	Lacto-ovo vegetarian	Pesco-vegetarian	Semi-vegetarian	Non-vegetarian	
Diabetes	26 [1.0]	356 [0.9]	46 [0.9]	48 [1.4]	71 [0.9]	1223 [1.2]	0.002
N [%], 95% CI	0.6-1.7	0.8-1.1	0.6-1.3	1.0-2.0	0.7-1.1	1.1-1.3	
BMI ≥23 kg/m^2^	534 [21.5]	9722 [26.9]	1163 [24.9]	650 [19.5]	1690 [21.8]	21380 [22.6]	<0.001
N [%], 95% CI	19.5-23.7	26.3-27.5	23.4-26.5	17.8-21.3	20.7-23.0	22.3-23.0	
BMI ≥25 kg/m^2^	286 [11.5]	5861 [16.2]	697 [14.9]	334 [10.0]	877 [11.3]	11996 [12.7]	<0.001
N [%], 95% CI	10.0-13.2	15.7-16.7	13.7-16.3	8.0-11.3	10.5-12.2	12.4-13.0	
BMI ≥30 kg/m^2^	58 [2.3]	1311 [3.6]	140 [3.0]	56 [1.7]	156 [1.6]	2269 [2.4]	<0.001
N [%], 95% CI	1.7-3.2	3.4-3.9	2.5-3.7	1.2-2.4	1.2-2.4	2.3-2.5	
BMI, mean [±SD]	20.5 [±4.2]	21.2 [±4.5]	21.0 [±4.1]	20.3 [±3.8]	20.6 [±4.0]	20.7 [±4.1]	

*P for trend values has been obtained from Likelihood ratio test for no difference between the groups for types of vegetarian diet ignoring the correlated data. As the non-vegetarian group was expected to have the highest and the rural group the lowest levels of diabetes and BMI, trend tests were carried out scoring the groups 1 to 5 and using likelihood ratio tests.

### Vegetarian diet consumption according to non-dietary variables

Table 5 shows the vegetarian diet consumption by non-dietary variables. Socioeconomic demographic and lifestyle characteristics differed substantially (p < 0.001) among the dietary groups but overall non-vegetarian diet was predominant in all socio economic and demographic categories followed by lacto-vegetarian diet.

**Table 5:** **Percentage distribution of non-dietary variables according to types of vegetarian diet consumption in adult Indian population (n = 156,317) aged 20–49 years, NFHS 2005-06**

Characteristics	Type of diets						Chi sq p values
	Vegan	Lacto-vegetarian	Lacto-ovo vegetarian	Pesco-vegetarian	Semi-vegetarian	Non-vegetarian	
Age							<0.001
20-29 y	1097 [1.7]	14846 [22.5]	2535 [3.8]	1234 [1.9]	3772 [5.7]	42553 [64.4]	
30-39 y	856 [1.6]	12758 [24.3]	1553 [3.0]	1203 [2.3]	2550 [4.9]	33648 [64.0]	
40-49 y	609 [1.6]	10196 [27.0]	913 [2.4]	1005 [2.7]	1817 [4.8]	23172 [61.4]	
Sex							<0.001
Men	406 [0.7]	10683 [18.8]	2226 [3.9]	955 [1.7]	3465 [6.1]	39009 [68.7]	
Women	2156 [2.2]	27118 [27.2]	2775 [2.8]	2487 [2.5]	4675 [4.7]	60364 [60.6]	
Education							<0.001
No education	1207 [2.1]	12896 [22.7]	1115 [2.0]	1363 [2.4]	3183 [5.6]	36957 [65.2]	
Primary	455 [1.9]	5645 [20.6]	599 [2.4]	628 [2.6]	1304 [5.3]	16462 [67.2]	
Secondary	755 [1.3]	14505 [24.8]	2199 [3.8]	1185 [2.0]	2967 [5.1]	36817 [63.0]	
Higher and above	125 [0.8]	5355 [32.2]	1087 [6.5]	265 [1.6]	686 [4.1]	9120 [54.8]	
Caste/tribe							<0.001
Scheduled caste	456 [1.6]	4343 [15.0]	836 [2.9]	731 [2.5]	2018 [7.0]	20545 [71.0]	
Scheduled tribe	311 [2.4]	1603 [12.6]	271 [2.1]	264 [2.1]	709 [5.6]	9576 [75.2]	
Other backward class	1019 [1.7]	16614 [27.2]	2160 [3.5]	1319 [2.2]	3026 [5.0]	36839 [60.4]	
General	761 [1.6]	15087 [30.9]	1692 [3.5]	1014 [2.1]	2110 [4.3]	28189 [57.7]	
Missing caste	9 [0.2]	109 [2.6]	31 [0.7]	92 [2.2]	222 [5.2]	3808 [89.2]	
Religion							<0.001
Hindu	2358 [1.9]	35337 [27.7]	4522 [3.6]	3069 [2.4]	6192 [4.9]	75897 [59.6]	
Muslim	78 [0.4]	272 [1.4]	196 [1.0]	260 [1.3]	1417 [7.2]	17558 [88.8]	
Christian	6 [0.2]	27 [0.7]	32 [0.8]	65 [1.7]	102 [2.7]	3585 [93.9]	
Sikh	75 [2.6]	1561 [54.9]	170 [6.0]	10 [0.4]	286 [10.1]	742 [26.1]	
Others	45 [1.8]	602 [24.1]	81 [3.2]	38 [1.5]	143 [5.7]	1591 [63.6]	
Wealth quintiles							<0.001
Lowest	586 [2.2]	4777 [18.1]	427 [1.6]	789 [3.0]	1051 [4.0]	18759 [71.1]	
Second	509 [1.8]	6225 [21.7]	735 [2.6]	741 [2.6]	1515 [5.3]	19026 [66.2]	
Middle	468 [1.5]	6768 [21.7]	846 [2.7]	725 [2.3]	1997 [6.4]	20428 [65.4]	
Fourth	516 [1.5]	7760 [23.1]	1167 [3.5]	667 [2.0]	1944 [5.8]	21506 [64.1]	
Highest	483 [1.3]	12270 [33.7]	1826 [5.0]	521 [1.4]	1633 [4.5]	19653 [54.0]	
Place of residence							<0.001
Urban	757 [1.4]	12685 [23.4]	2259 [4.2]	904 [1.7]	2964 [5.5]	34565 [63.9]	
Rural	1804 [1.8]	25116 [24.6]	2741 [2.7]	2538 [2.5]	5176 [5.1]	64808 [63.4]	
Current Tobacco smoking							<0.001
No	2388 [1.8]	34150 [25.6]	4258 [3.2]	3026 [2.3]	6687 [5.0]	82651 [62.1]	
Yes	173 [0.7]	3651 [15.8]	742 [3.2]	416 [1.8]	1453 [6.3]	16721 [72.2]	
Alcohol consumption							<0.001
Never	2496 [1.9]	36605 [27.5]	4320 [3.2]	3102 [2.3]	6707 [5.0]	79745 [60.0]	
Ever	66 [0.3]	1196 [5.1]	681 [2.9]	339 [1.5]	1431 [6.1]	19611 [84.1]	
Frequency of watching TV							<0.001
Not at all	1177 [2.6]	12046 [26.2]	937 [2.0]	1269 [2.8]	2170 [4.7]	28318 [61.7]	
Less than once a week	245 [1.1]	4789 [21.9]	588 [2.7]	467 [2.1]	884 [4.0]	14886 [68.1]	
At least once a week	314 [1.6]	4639 [23.2]	663 [3.3]	476 [2.4]	1128 [5.6]	12814 [64.0]	
Almost everyday	824 [1.2]	16322 [23.8]	2812 [4.1]	1230 [1.8]	3956 [5.8]	43336 [63.3]	
Total	2560 [1.6]	37797 [24.2]	5002 [3.2]	3446 [2.2]	8140 [5.2]	99372 [63.6]	156317

### Association between type of vegetarian diet and diabetes

In multivariable logistic regression analysis (Table 6), after adjustment for age, gender, education, household wealth, rural/urban residence, religion, caste, smoking, alcohol use, television watching and body mass index, consumption of lacto-vegetarian (AOR:0.67; 95% CI:0.58-0.76), lacto-ovo vegetarian (AOR:0.69; 95% CI:0.50-0.95) and semi-vegetarian (AOR:0.76; 95% CI: 0.60-0.98) diets were associated with a lower likelihood of diabetes than non-vegetarian diet. The association remained almost unchanged when BMI was removed from the analyses.

**Table 6:** **Multivariable logistic regression analysis (odds ratio with 95**% **confidence interval) of the relation between types of vegetarian diet and self reported diabetes, in adult Indian population aged 20–49 years, NFHS-3 2005-06**

Characteristics	Unadjusted OR [95% CI]	P values	Adjusted* OR [95% CI]	P values	Adjusted ψ OR [95% CI]	P values
Types of vegetarian diet						
Non-vegetarian (ref)	1		1		1	
Semi-vegetarian	0.71 [0.56-0.90]	0.005	0.77 [0.60-0.98]	0.034	0.76 [0.59-0.96]	0.024
Pesco-vegetarian	1.13 [0.84-1.51]	0.426	1.15 [0.85-1.54]	0.365	1.09 [0.81-1.46]	0.589
Lacto-ovo vegetarian	0.74 [0.55-1.00]	0.047	0.70 [0.51-0.96]	0.025	0.73 [0.54-0.99]	0.044
Lacto-vegetarian	0.76 [0.68-0.86]	<0.001	0.67 [0.58-0.76]	<0.001	0.66 [0.58-0.75]	<0.001
Vegan	0.81 [0.55-1.20]	0.289	0.91 [0.61-1.36]	0.643	0.89 [0.59-1.32]	0.553
Age						
20-29y (ref)	1		1		1	
30-39y	3.23 [2.74-3.80]	<0.001	2.83 [2.39-3.35]	<0.001	3.32 [2.82-3.91]	<0.001
40-49y	9.20 [7.91-10.69]	<0.001	7.78 [6.64-9.12]	<0.001	9.39 [8.05-10.95]	<0.001
Sex						
Men (ref)	1		1		1	
Women	0.83 [0.75-0.91]	<0.001	0.87 [0.76-0.99]	0.029	0.94 [0.83-1.06]	0.298
Education						
No education (ref)	1		1		1	
Primary	1.46 [1.27-1.69]	<0.001	1.20 [1.03-1.40]	0.022	1.23 [1.060-1.44]	0.008
Secondary	1.53 [1.36-1.71]	<0.001	1.22 [1.05-1.41]	0.008	1.28 [1.11-1.47]	0.001
Higher and above	1.63 [1.39-1.91]	<0.001	1.05 [0.85-1.28]	0.672	1.11 [0.91-1.36]	0.300
Caste/tribe						
Scheduled caste (ref)	1		1		1	
Scheduled tribe	0.40 [0.30-0.54]	<0.001	0.47 [0.34-0.63]	<0.001	0.47 [0.35-0.63]	<0.001
Other backward class	0.98 [0.85-1.12]	0.714	0.84 [0.73-0.98]	0.022	0.86 [0.75-0.99]	0.039
General	1.38 [1.21-1.58]	<0.001	0.95 [0.81-1.10]	0.459	0.98 [0.84-1.13]	0.751
Missing caste	1.61 [1.26-2.06]	<0.001	1.18 [0.90-1.55]	0.244	1.20 [0.91-1.59]	0.199
Religion						
Hindu (ref)	1		1		1	
Muslim	1.26 [1.11-1.44]	<0.001	1.14 [0.98-1.33]	0.100	1.16 [0.99-1.35]	0.062
Christian	1.85 [1.46-2.33]	<0.001	1.31 [1.02-1.68]	0.035	1.36 [1.07-1.74]	0.014
Sikh	1.25 [0.91-1.73]	0.173	0.85 [0.61-1.19]	0.333	0.96 [0.69-1.33]	0.787
Others	0.83 [0.54-1.26]	0.375	0.70 [0.44-1.10]	0.119	0.74 [0.48-1.13]	0.159
Wealth quintiles						
Lowest (ref)	1		1		1	
Second	1.56 [1.27-1.92]	<0.001	1.40 [1.13-1.73]	0.002	1.42 [1.15-1.76]	0.001
Middle	1.38 [1.12-1.71]	0.002	1.05 [0.84-1.32]	0.654	1.16 [0.93-1.44]	0.201
Fourth	2.39 [1.98-2.90]	<0.001	1.53 [1.22-1.93]	<0.001	1.80 [1.44-2.25]	<0.001
Highest	3.77 [3.15-4.51]	<0.001	1.87 [1.45-2.41]	<0.001	2.50 [1.96-3.19]	<0.001
Place of residence						
Urban	1.96 [1.79-2.16]	<0.001	1.20 [1.07-1.35]	0.002	1.24 [1.10-1.39]	<0.001
Rural (ref)	1		1		1	
Body Mass Index (kg/m^2^)						
≤18.5 kg/m^2^	0.56 [0.49-0.64]	<0.001	0.75 [0.65-0.88]	<0.001	-	
18.5-22.9 kg/m^2^ (ref)	1		1			
23.0-24.9 kg/m^2^	2.57 [2.26-2.93]	<0.001	1.64 [1.42-1.91]	<0.001	-	
≥25.0 kg/m^2^	4.06 [3.65-4.52]	<0.001	2.21 [1.95-2.51]	<0.001	-	
Current tobacco smoking						
No (ref)	1		1		1	
Yes	1.13 [0.99-1.28]	0.069	0.98 [0.84-1.15]	0.803	0.93 [0.80-1.09]	0.379
Alcohol consumption						
Never (ref)	1		1		1	
Ever	1.15 [1.01-1.30]	0.029	1.01 [0.87-1.19]	0.870	1.05 [0.90-1.23]	0.519
Frequency of watching TV						
Not at all (ref)	1		1		1	
Less than once a week	1.10 [0.92-1.31]	0.307	0.89 [0.74-1.07]	0.202	0.92 [0.76-1.10]	0.345
At least once a week	1.34 [1.13-1.58]	0.001	0.95 [0.79-1.14]	0.594	1.00 [0.84-1.20]	0.975
Almost everyday	1.84 [1.63-2.08]	<0.001	0.91 [0.78-1.07]	0.258	1.04 [0.89-2.21]	0.652

*Adjusted for all factors; Ψ Adjusted for all factors except BMI; OR- indicates odds ratios; ref- indicates reference category.

### Association between type of vegetarian diet and diabetes stratified by sex

To examine the sex differences in the adjusted effect of vegetarian diet on diabetes prevalence, we also carried out separate analyses for men and women (Table 7). The likelihood of having a positive diabetes status was significantly lower among men following a lacto-vegetarian (AOR:0.66; 95% CI:0.52-0.82;p < 0.0001) and semi-vegetarian diet (AOR:0.45; 95% CI:0.29-0.71;p = 0.001) while only lacto-vegetarian diet (AOR:0.70; 95% CI:0.59-0.82;p < 0.0001) consumption was associated with a lower likelihood of diabetes among women.

**Table 7:** **Multivariable logistic regression analysis (odds ratio with 95**% **confidence interval) of the relation between types of vegetarian diet and self reported diabetes, in men (n = 56742) and women (n = 99574) aged 20–49 years, NFHS-3 2005-06**

Predictors	Unadjusted OR [95% CI]	P values	Adjusted* OR [95% CI]	P values	Adjusted Ψ OR [95% CI]	P values
Men						
Types of vegetarian diet						
Non-vegetarian (ref)	1		1		1	
Semi-vegetarian	0.47 [0.31-0.72]	<0.001	0.45 [0.29-0.71]	0.001	0.48 [0.32-0.73]	0.001
Pesco-vegetarian	0.78 [0.42-1.44]	0.426	0.80 [0.43-1.50]	0.488	0.77 [0.41-1.43]	0.407
Lacto-ovo vegetarian	0.74 [0.49-1.13]	0.165	0.63 [0.39-1.00]	0.050	0.72 [0.47-1.11]	0.134
Lacto-vegetarian	0.79 [0.64-0.96]	0.020	0.66 [0.52-0.82]	<0.001	0.65 [0.52-0.81]	<0.001
Vegan	0.67 [0.24-1.85]	0.438	0.70 [0.25-1.96]	0.498	0.66 [0.24-1.83]	0.424
Body Mass Index (kg/m^2^)						
≤18.5 kg/m^2^	0.75 [0.60-0.93]	0.009	0.85 [0.68-1.06]	0.147	-	
18.5-22.9 kg/m^2^ (ref)	1		1		-	
23.0-24.9 kg/m^2^	2.29 [1.85-2.83]	<0.001	1.63 [1.30-2.03]	<0.001	-	
≥25.0 kg/m^2^	3.06 [2.53-3.71]	<0.001	1.80 [1.46-2.23]	<0.001	-	
Women						
Types of vegetarian diet						
Non-vegetarian (ref)	1		1		1	
Semi-vegetarian	0.92 [0.68-1.23]	0.561	1.09 [0.81-1.47]	0.582	1.03 [0.77-1.39]	0.842
Pesco-vegetarian	1.33 [0.96-1.86]	0.090	1.33 [0.95-1.87]	0.101	1.24 [0.88-1.74]	0.220
Lacto-ovo vegetarian	0.72 [0.47-1.10]	0.128	0.77 [0.50-1.19]	0.238	0.75 [0.49-1.14]	0.176
Lacto-vegetarian	0.78 [0.67-0.90]	0.001	0.70 [0.59-0.82]	<0.001	0.69 [0.59-0.81]	<0.001
Vegan	0.89 [0.58-1.37]	0.606	1.01 [0.65-1.56]	0.984	0.98 [0.64-1.52]	0.931
Body Mass Index (kg/m^2^)						
≤18.5 kg/m^2^	0.60 [0.49-0.74]	<0.001	0.68 [0.56-0.84]	<0.001	-	
18.5-22.9 kg/m^2^ (ref)	1		1		-	
23.0-24.9 kg/m^2^	2.23 [1.83-2.71]	<0.001	1.63 [1.34-2.00]	<0.001	-	
≥25.0 kg/m^2^	4.32 [3.72-5.01]	<0.001	2.44 [2.07-2.87]	<0.001	-	

*Adjusted for age, education, caste/tribe, religion, wealth quintiles, place of residence, BMI, current tobacco smoking, alcohol consumption and frequency of watching TV. Ψ Adjusted for all factors except BMI. OR: odds ratios.

ref- reference category.

## Discussion

This cross-sectional, population-based study adds to the limited data on associations between type of vegetarian diet intake and diabetes prevalence in developing countries. Our finding suggest that persons consuming a lacto vegetarian, lacto-ovo vegetarian or semi-vegetarian diet had a lower likelihood of diabetes compared with those consuming non-vegetarian diet after adjustment for a number of socioeconomic and lifestyle factors. These findings may be explained by adverse effects of meat and fish, protective effects of typical constituents of lacto-vegetarian and lacto-ovo vegetarian diet which have been demonstrated elsewhere [23–26]. Our study indicates that the body mass index of Indian vegetarian diet consumers did not differ significantly from their non-vegetarian counterparts, but the male vegetarians appeared to be significantly (p < 0.0001) uniformly leaner than female vegetarians. This association between vegetarianism and non-leanness is in line with a health study among the Barbados Seventh-Day Adventists which found self-reported vegetarians of less than 5 years did not differ significantly from the non-vegetarians [27].

Our results are in consistent with those of previous studies among various seventh-Day Adventist churchgoers [1, 2, 5, 6, 28, 29], several other studies conducted in western countries [10, 30–37] and an Indian study [38] which showed that increased conformity to vegetarian diets is protected against risk of type 2 diabetes and hypertension. Findings from an accumulating number of studies have also shown evidence that most vegetarian diets are not only nutritionally adequate but also associated with lower risk of certain chronic diseases, when compared with the effects of a more typical western diets [36]. Evidence from a number of other observational studies shows that certain dietary constituents are associated with protection against diabetes through the pathway of insulin sensitivity which is also confirmed through food trials [10]. A reduced risk of chronic disease has been reported in populations of vegetarians living in affluent countries [39–41] and in case–control comparisons in developing countries [42]. Reduced consumption of animal fat and increased consumption of fruit, vegetables, foods that have a low glycemic index such as beans, legumes, nuts and cereals including whole grain and foods that reduce oxidative stress and chronic inflammation [2] may underlie such a protective effect. Whole grain is also a potential contributor to reduced diabetes risk in vegetarians and accumulating evidences from various prospective studies (both in men and women) and several meta-analyses shows that consumption of whole grains may reduce risk of chronic diseases including type 2 diabetes [43–49]. A recent meta-analysis [49] shows that the summary relative risk per 3 servings per day was 0.68 (95%CI:0.58–0.81;I^2^ = 82%;n = 10) for whole grains and 0.95 (95%CI:0.88–1.04;I^2^ = 53%;n =6) for refined grains. In addition to the influence of fiber and glycemic load on postprandial glucose and insulin response [50], whole grains may also reduce the risk of type 2 diabetes through the action of nutrients such as vitamin E and magnesium [50–52].

We did not find an expected association between vegans (who eat no animal products) and significant reduced diabetes prevalence in our study contrary to other western studies. The term ‘vegan’ which may not be correctly asked/interpreted/self-reported in Indian context might be an important reason for this unexpected association, as because vegans in western countries necessarily do not have any form of dairy, including butter or *ghee* or any animal products, including honey which might not be the case for Indian vegans; self-reported vegans in India probably have butter/*ghee*/honey in their diet. Second, it may be likely that vegans may be eating more of the refined-rice diets than non-vegetarians and unlike in the West, vegans in India would not be doing it for health-conscious/political reasons. Also, since this is a cross-sectional study, we may have some reverse casuality –diabetes patients changing to a vegan diet after the diabetes diagnosis. Another issue could be the statistical power. We found the odds ratio in vegan men is very similar to that in lacto-vegetarian men, while the result in women is null. Thus, the small sample size in the vegan group, which constituted only 1.6% of the sample, might have influenced the study results. However, though the more recent official statements from American Diabetes Association has clearly described vegetarian diets as healthful [53] and some studies [1, 2] shows that vegans have a least risk of type 2 diabetes, still the association between vegan diet and diabetes risk is open to question.

The notion that animal protein stimulates insulin secretion and possibly insulin resistance was proposed decades ago [54]. Red and processed meat consumption has been associated with increased risk of type 2 diabetes in a large number of cohort studies in the west [6, 55–57]. Meat intake was associated with a higher risk of diagnosed diabetes in a study in Seventh-Day Adventists [8]. Several other studies around the globe have also reported an increased risk of diabetes or type 2 diabetes with a higher intake of processed meat [7, 9, 58–62], red meat [7, 58, 59, 62–64] and total meat [7, 9, 65], but in some studies the results have been inconsistent [55, 61].

The categories that we have used to distinguish different types of diet in our study is internationally recognized [2, 8, 17, 18] and have also proven to be categories that have markedly different risks of common diseases such as diabetes and hypertension in cohort [1, 2, 66] and observational studies in UK [37] but it is possible that the more-refined categories may provide better comparability.

The strengths of our study include the use of large nationally representative study sample which allows comparisons to be made between men and women and the ability to examine this association in adult Indian population. Also rigorous efforts were made in the NFHS-3 to obtain reliable self-reported data: the survey used local terminology and commonly understood terms to describe the disease, rigorously trained interviewers, supervisors and standard quality checks [16] (also see http://www.dhsprogram.com).

The prevalence of diabetes in this large nationally representative survey was comparatively low (1.1%) than studies conducted in selected geographical areas or cities in India [11, 14, 67–71]. The low diabetes prevalence in our study reflects the young age of this population, the use of self-reports rather than biochemical assessments and sampling from the general population that included a high proportion of respondents in rural areas [72]. Our study has added to this literature using a national population health survey with good coverage in rural areas across India. Estimates from a recent study of rural–urban migrants showed an age-adjusted prevalence of diabetes (diagnosed using both self-reports and fasting blood glucose in relatively affluent populations) of 10–15% in urban people and 5–6% in rural people of similar age to those recruited in NFHS-3 [73]. In most urban parts of India the health system is well enough developed for diagnosis of symptomatic diabetes, but at younger ages (<30 years) diabetes may not be symptomatic and NFHS-3 prevalence estimates are undoubtedly conservative, particularly for rural India where diagnosis may be much less likely to occur [74]. However, this ascertainment bias is unlikely to have been differential with respect to types of vegetarian diet consumption. In other words, although we clearly have sub-set of disease, it’s unlikely to be systematically different from entire group in terms of dietary patterns.

Previous research has shown a good agreement for self-reported diabetes when compared with medical records in a US population [75] and that self-reported health conditions demonstrate the expected relationship with socioeconomic status in India [76]. Studies in India also have shown that the difference between self reports and objective measurements according to education and awareness levels does not preclude the use of self-reports [72, 76]. On the contrary to the prevailing view that there is a positive (or null) association between measures of socio economic status and self-reported poor health/morbidities in less-developed countries, and that any potential “under-reporting” is not only smaller than the difference in prevalence of illness between the socially disadvantaged and the advantaged, the study by Subramanian et al. [76] showed that the same is even true within groups with the same objective diagnosis. In addition, our analyses considering respondents who reported ‘unknown’ for diabetes status were nearly identical to the main analyses (data not shown). Although our sample was relatively young (<50 years for women and men both), it is representative of the young population profile of India; 84% of the Indian adult population (18–69 years) and 47% of the total Indian population at all ages fall within the ages covered by this study [77]. Our study does exclude approximately 14% of the Indian population (men and women over age 50) due to the sample design of the NFHS. The prevalence of diabetes increases with age and whether a similar socioeconomic status–diabetes relationship exists among middle and older age groups in all parts India is not clear [72], although our findings are consistent with the previous studies that have included older ages.

The current national estimate for diabetes prevalence in India is about 7% of the adult population aged 20–79 years [72]; estimates being based on three relatively recent and large scale studies using a combination of oral glucose tolerance testing and self-reports of diabetes [11, 78]. There continues to be considerable uncertainty in estimates of diabetes for the whole of India due to the limited study locations (with a focus on urban areas), wide variation in survey sampling methodology, differences in diabetes diagnostic criteria and age groups studied [72]. These differences in study design have hindered direct comparison of the prevalence between studies, across regions and over time. The NFHS-3 provides an important benchmark because it is the first nationally representative survey of diabetes in India. Even if the prevalence estimates of diabetes have been underestimated in the NFHS-3, the observed diet–diabetes associations are reasonable and significant, and can be comparable to cohort and prospective studies on similar association in the west. Previous studies have largely overlooked the importance of modifiable dietary factors, which may be a key determinant of diabetes in Indians, given the varied nature of Indian diets. Further large-scale population-based surveys can be strengthened by using simple finger-prick blood glucose measurements in addition to self-reports.

In our analyses, the cross-sectional design precludes causal inferences and we were limited to the questions used to elicit lifestyle and dietary information. Given the high proportion of undiagnosed diabetes in developing countries including India (http://www.worlddiabetesfoundation.org) where less than half of people with diabetes are diagnosed, there is a possibility that the exposure was associated with the likelihood of testing for diabetes, which may result in detection bias. Importantly the entire study may be with known diabetic subjects who would have altered diet and hence might have increased or decreased vegetarian diet consumption due to the dietary advice based on diabetes control and complications of diabetes like nephropathy. General dietary advice given to diabetic subjects is to include more whole grains, legumes, fruits and green leafy and other vegetables as this is evident in our data where more than 90% of the self-reported diabetics did report ‘daily’ or ‘weekly’ consumption of legumes, vegetables and fruits–all suggest that the dietary choices of self-reported diabetic subjects might have been modified to manage diabetes. However, despite these shortcomings rigorous precautions were taken in the NFHS to obtain reliable self-reported data such as the survey used the local terminology and commonly understood term of the disease, rigorously trained interviewers and supervisors and standard quality checks.

Nevertheless, our study has some other limitations. Misclassification of dietary information, although unavoidable, would most likely be non-differential and thus may attenuate the true association. There were relatively small numbers in some of the dietary categories, which should be considered when interpreting the findings in relation to these diets. There might be limitation of the dietary assessment method in NFHS-3 as well since there may be other foods that are associated with diabetes that are not asked to the respondents. We were also unable to distinguish between Type 1 and 2 diabetes diagnoses. Since the NFHS-3 questionnaire is interviewer administered, information on the inter rater compatibility, reproducibility and validity of questionnaire would be critical to evaluate the ability of such questionnaire to measure true dietary intake. But NFHS-3 being a part of Demographic and Health Surveys (available at http://www.dhsprogram.com) which is conducted in more than 80 countries with similar questionnaire seems to be fairly valid to get an overall picture of frequency of dietary intake in a population [79]. However, under- and over-reporting could lead to a biased estimation of the association between dietary factors and diabetes. Although we adjusted for several confounding variables, we cannot exclude the possibility of residual confounding. However, if this was the case, similar effects would be expected for dietary components that are related to greater affluence, which was not observed.

Another limitation of our study is reliance on self-reports of diabetes which has resulted in a marked underestimation of prevalence, and its focus on people <60 years in whom diabetes is less common [74]. Self-reported data, especially in rural areas, can be flawed owing to several factors such as lack of awareness, low educational status, limited access to health services and hesitation to disclose diagnosed diseases. But in developing countries, self-reporting should not be a prohibitive limitation as medically diagnosed and/or biomarker-confirmed prevalence estimates are nearly impossible for nationwide prevalence estimates in low- resource and low-access settings such as India. Also since, the low and middle income countries has only very limited nutrition and health outcomes data, NFHS-3 is therefore the best available dataset to examine the relationship.

Valid data on physical activity were not available in NFHS-3 which is a limitation of this study since persons with healthier diets may be physically more active than other persons [80]. Therefore the lack of physical activity data may have confounded the results. Moreover, assessment of sedentary habits in this study was based on hours of TV watching. However, physical activity has in part been accounted for, indirectly, by adjusting for body mass index. In the present study, adjustment for socioeconomic and demographic factors, residential location, religion and caste/tribe status of the respondents did not markedly modify the adjusted result, suggesting that the associations are not completely explained by non-dietary lifestyle factors. Further studies are needed to determine whether the association between diet and diabetes is mediated by assumed nutrients or by lifestyle and socioeconomic and demographic factors related to frequency of food consumption.

The rising burden of diabetes in India requires a rapid response that integrates policies and programmes which enable effective prevention and control across diverse geographical and low-resource settings. Our findings on inverse association between types of vegetarian diet consumption and diabetes prevalence can be considered by policy-makers to promote healthy vegetarian diet consumption in Indian population and to discourage unhealthy non-vegetarian diets. There is, therefore, an opportunity to modify the direction and dimensions of this national epidemic through policy interventions (at the state level), which promote the availability, affordability and acceptability of vegetarian diets more specifically lacto vegetarian and lacto-ovo vegetarian diets and restrain the marketing and consumption of unhealthy non-vegetarian foods. This requires coordinated action at the level of governments, civil society and responsible sections of the food industry.

## Conclusions

In conclusion, our findings are important for public health interventions in diabetes care in India which shows that, in a large sample of adult men and women in India, variants of vegetarian diets such as lacto-vegetarian and lacto-ovo vegetarian were associated with at least a 30% lower risk of diabetes. These results add to the limited evidence in developing countries that shows potential benefits of consuming vegetarian diets to reduce the development of diabetes. These findings need further validation by longitudinal and clinical studies but may well have public health significance for the Indian population. These findings, if replicated using objective and comprehensive methods of dietary intake and diabetes, may inform the development of interventions to address the growing burden of overweight and diabetes in India.

## Endnotes

^a^The scale has a 200 kg capacity and weighs in 0.01 kg increments. The scale is powered by six AA batteries and has an “ON-OFF” switch located at the side of the scale. The SECA 874 digital floor scale is manufactured by Seca gmbh & co. kg. Hammer Steindamm 9 – 25. 22089 Hamburg. Germany. The scale can be procured directly from Seca. These instructions were adapted from instructions that accompany the scale and revised by Irwin J. Shorr, MPH, MPS.

^b^The state of a person’s health in terms of the nutrients in his or her diet. In Indian context, it also means inadequate and poor diet and repeated exposure to disease and illness, may or may not be based on any clinical test or measurement.
